# Human T-Cell Leukemia Virus Type 1 Envelope Protein: Post-Entry Roles in Viral Pathogenesis

**DOI:** 10.3390/v14010138

**Published:** 2022-01-13

**Authors:** Victoria Maksimova, Amanda R. Panfil

**Affiliations:** 1 Biomedical Sciences Graduate Program, Center for Retrovirus Research, Department of Veterinary Biosciences, College of Veterinary Medicine, The Ohio State University, Columbus, OH 43210, USA; maksimova.1@osu.edu; 2 Center for Retrovirus Research, Comprehensive Cancer Center and Solove Research Institute, Department of Veterinary Biosciences, College of Veterinary Medicine, The Ohio State University, Columbus, OH 43210, USA

**Keywords:** HTLV-1, HTLV-2, envelope, transformation, CD4^+^ T-cell, ATL, CD8^+^ T-cell

## Abstract

Human T-cell leukemia virus type 1 (HTLV-1) is an oncogenic retrovirus that is the causative infectious agent of adult T-cell leukemia/lymphoma (ATL), an aggressive and fatal CD4^+^ T-cell malignancy, and HTLV-1-associated myelopathy/tropical spastic paraparesis (HAM/TSP), a chronic neurological disease. Disease progression in infected individuals is the result of HTLV-1-driven clonal expansion of CD4^+^ T-cells and is generally associated with the activities of the viral oncoproteins Tax and Hbz. A closely related virus, HTLV-2, exhibits similar genomic features and the capacity to transform T-cells, but is non-pathogenic. In vitro, HTLV-1 primarily immortalizes or transforms CD4^+^ T-cells, while HTLV-2 displays a transformation tropism for CD8^+^ T-cells. This distinct tropism is recapitulated in infected people. Through comparative studies, the genetic determinant for this divergent tropism of HTLV-1/2 has been mapped to the viral envelope (Env). In this review, we explore the emerging roles for Env beyond initial viral entry and examine current perspectives on its contributions to HTLV-1-mediated disease development.

## 1. Introduction

Human T-cell leukemia virus type 1 (HTLV-1), the first human retrovirus discovered [1], is highly endemic to regions such as Southwestern Japan, Sub-Saharan Africa, South America, and the Caribbean, with clusters of infection in the Middle East and Australo-Melanesia [2]. While there are an estimated 5–10 million HTLV-1-infected individuals worldwide, large population-based studies are still lacking, and previous analyses have focused on specific groups, including blood donors, pregnant women, and other high-risk patients [2]. It is therefore likely that the number of virus-infected carriers is higher than the current estimate. Transmission of HTLV-1 occurs along three major routes: mother-to-child via breastfeeding or childbirth, sexual contact, or exposure through infected blood (i.e., blood transfusion, intravenous drug use, organ transplantation) [3,4,5,6,7]. In regions of viral endemicity, infection is primarily transmitted to infants through breast milk, with increased rates of seroconversion corresponding to the duration of breastfeeding [8,9,10]. Japan, which has areas of high HTLV-1 prevalence, has offered pregnant women serological screening for HTLV-1 antibodies since 2010 [11] and guidance to exclusively bottle-feed, freeze–thaw breast milk, or cease breastfeeding at 3 months—except in high-risk cases, such as with premature infants [12]. Although a nationwide strategy to prevent vertical transmission has been implemented successfully in Japan [13], there are no universal precautions for HTLV-1 infection with breastfeeding, and the current recommendations may be impractical in developing countries due to socio-economic barriers [14]. Furthermore, while rare (less than 3% of cases), transmission of the virus is possible transplacentally or during delivery [15]. In addition, there are few established programs to reduce HTLV-1 sexual transmission, and this contributes to the enduring prevalence of infection in Japan [16] and other regions such as South America [17,18], as well as the rising incidence outside of typical endemic areas. A recent epidemic was discovered in an indigenous population of central Australia, where adult seropositivity rates surpassed 40% and were associated with increased age, gender, and history of sexually transmitted infections [19,20]. Lastly, systematic screening of donated blood and organs is not conducted in many countries where HTLV-1 is endemic [13,21], and seroconversion rates from 44–63% have been documented in recipients of HTLV-1-positive blood products [4,22]. Until routine testing of donors for HTLV-1 began in the United States in 1988, transfusion of infected blood correlated with a seroconversion rate of 12.8%, with a sharp increase to 80% if blood products were transfused within five days of donation [23]. 

HTLV-1 is the causative infectious agent of adult T-cell leukemia/lymphoma (ATL), an aggressive and fatal CD4^+^ T-cell malignancy [1,24,25] that develops in approximately 5–10% of infected individuals after a long clinical latency period that spans upwards of five decades [26]. Viral infection can also cause HTLV-1-associated myelopathy/tropical spastic paraparesis (HAM/TSP) [27,28], a neurological disorder characterized by a chronic inflammatory response against HTLV-1-infected CD4^+^ T-cell infiltrates in the central nervous system [29]. Other diseases associated with HTLV-1 infection, including uveitis and infective dermatitis, involve chronic inflammation against HTLV-1-infected CD4^+^ T-cells [30]. ATL is categorized into four diverse clinical subtypes (smoldering, chronic, lymphoma, and acute) [31] that dictate patient prognosis and treatment strategies [30]. For the most aggressive ATL subtypes, acute and lymphoma, the median survival time is less than 1 year—even with treatment [31]. Factors such as a large tumor burden and a lack of efficacious therapeutic drugs pose major challenges to the treatment of ATL [30]. A nationwide prospective study of asymptomatic HTLV-1 carriers in Japan determined that risk factors for ATL progression include high proviral load (>4 copies/100 peripheral blood mononuclear cells), a family history of ATL, older age, and the first opportunity for HTLV-1 testing occurring at the time of treatment for other conditions [32]. A feature often used for the diagnosis of ATL is the presence of abnormal T-cells called ‘flower’ cells in the peripheral blood. These abnormal cells, which have multilobulated nuclei with condensed chromatin and discreet nucleoli, frequently make up more than 5% of circulating T-cells in patients with histologically undetectable tumor lesions [31,33].

HTLV-1 is classified within the deltaretrovirus genus. This genus encompasses the primate T-lymphotropic viruses, including the strains that infect humans (HTLVs) and bovine leukemia virus [34]. The single-stranded positive-sense viral RNA genome is composed of 8.5 kilobases encoding the structural and enzymatic genes present in all retroviruses (*gag*, *pro*, *pol*, and *env*), as well as regulatory and accessory genes from a unique region termed ‘pX’, located 3′ to the *env* gene [35]. HTLV-1 also encodes a gene on the viral antisense genome strand termed *hbz*. In newly infected cells, viral RNA is reverse transcribed into double-stranded DNA, which randomly integrates into the cellular genome. After integration, *tax/rex* is the initial dominant transcript that is produced through splicing of viral mRNA. Of the HTLV-1 gene products, at least two, *tax* and *hbz*, have been strongly implicated in T-cell transformation and ATL pathogenesis [36,37]. Tax amplifies viral transcription through the recruitment of cellular transcription factors to the viral promoter in the 5′ long-terminal repeat (LTR). In addition to its role in HTLV-1 transcription, Tax is also a potent modulator of cellular gene expression through the activation of the NF-κB and AP-1 signaling pathways [38]. Tax initiates cellular transformation through a variety of mechanisms; however, transformed ATL cells frequently lose Tax expression [39]. In contrast, Hbz is ubiquitously expressed in HTLV-1-infected cells and ATL patients [39,40,41]. It has been shown that both *hbz* mRNA and protein support the proliferation of infected cells and ATL cells in vitro [41,42], and that in vivo Hbz expression promotes viral persistence and leukemogenesis [43,44]. Although Tax is the primary driver of transformation, it is highly immunogenic [45], and thus its expression is tightly regulated to avoid immune recognition—whereby Hbz functions in its absence to maintain T-cell proliferation, survival, and persistent viral infection. 

While Tax and Hbz are key contributors to HTLV-1-mediated disease development, a potential pathogenic role for the viral envelope (Env) has emerged from comparative studies between HTLV-1 and HTLV-2. These viruses share high genomic sequence homology and functional similarity of their gene products [46,47], but differ in their pathogenesis. HTLV-2 has not been definitively linked to significant disease, though it was originally discovered in a patient with variant hairy cell leukemia of CD8^+^ T-cell origin, and a few cases of neurological conditions with HTLV-2 infection have been reported [48,49,50]. Compared to HTLV-1, the prevalence of HTLV-2 infection is much lower and generally limited to specific subpopulations. There are an estimated 800,000 HTLV-2-infected individuals globally, with a significant proportion of infections (400,000–500,000) found in Native American groups and intravenous drug users in the United States. The remainder is largely concentrated in Brazil, with similar epidemiological determinants [51]. The distinct clinical outcomes of HTLV-1 and HTLV-2 infection, despite similarities in the genetic organization and replication cycles of these viruses, have spurred numerous studies aiming to isolate the unique features driving HTLV-1-associated disease. This review will highlight the potential functions of Env beyond initial viral entry and infection in the context of cellular transformation studies involving HTLV-1 and HTLV-2.

## 2. Env-Mediated Viral Entry

HTLV-1 and HTLV-2 are transmitted mainly through cell-to-cell contact [52,53,54,55] and require viral Env glycoproteins to enable their binding and entry into target cells. While myeloid and plasmacytoid dendritic cell subsets have been shown to internalize HTLV-1 cell-free virions and become infected [56], cell-free infection by HTLV-1 and HTLV-2 is extremely inefficient [54,57,58,59,60]. The primary mechanisms for infection via cell-to-cell contact involve the formation of a virological synapse (VS), driven by the polarization of the microtubule organizing center of an infected cell toward a target cell [61,62]. This polarization is triggered by virus-induced upregulation of intercellular adhesion molecule 1 (ICAM-1) on the infected cell and its interaction with lymphocyte function-associated antigen (LFA-1) on the target cell [52,63,64]. The accumulation of Env and other viral proteins, including p19 matrix and p15 nucleocapsid, as well as viral genomes, has been found at this cell–cell interface; therefore, it is thought that viral assembly is coordinated with VS formation and transmission [52,61]. Furthermore, within the VS, HTLV-1 viral proteins, including Gag and Env, have been found to be concentrated in extracellular assemblies akin to a biofilm. These structures, composed of carbohydrates, extracellular matrix components, and cellular lectins, harbor clusters of virus particles and can be transferred from cell to cell to facilitate viral transmission [61,65]. Recent data have shown that adhesion molecules such as ICAM-1 and LFA-1, as well as the cluster of differentiation glycoproteins involved in T-cell migration, T-cell receptor signaling, and apoptosis (CD45 and CD43), are upregulated in extracellular vesicles (EVs) secreted by HTLV-1-infected cells [66]. These EVs have also been shown to package viral cargo, including the Gag-Pro-Pol precursor polyprotein, Env, and Tax [67].

**Figure 1:**
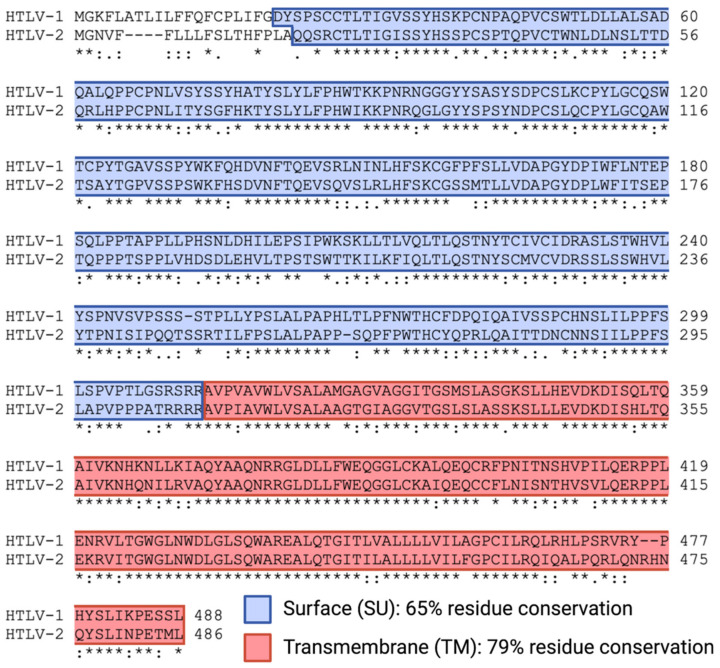
Amino acid sequence alignment of the HTLV-1 and HTLV-2 envelope proteins. The alignment was performed with the Clustal Omega program using the following UniProtKB accession numbers: P03381 for HTLV-1 Env and P03383 for HTLV-2 Env. The regions highlighted in blue and red correspond to the surface (SU) and transmembrane (TM) glycoproteins, respectively. Asterisks indicate positions with a fully conserved residue, colons indicate conservation between groups of strongly similar properties (scoring > 0.5 in the Gonnet PAM 250 matrix), and periods indicate conservation between groups of weakly similar properties (scoring ≤ 0.5 in the Gonnet PAM 250 matrix).

Env is synthesized as a precursor protein (gp62) that undergoes a maturation process in the endoplasmic reticulum, involving protein folding, oligomerization, and glycosylation [68]. When this precursor is transported through the Golgi, it is cleaved by cellular proteases to generate the surface (SU; gp46) and transmembrane (TM; gp21) glycoproteins [68]. These subunits are organized into trimers which remain associated through non-covalent linkages, where SU is found extracellularly and TM is embedded in the cell membrane or viral envelope [68]. SU and TM function in concert to facilitate viral entry; SU interacts directly with cell surface receptors and TM enables fusion of the viral and cellular membranes [69]. HTLV-1 Env (Env-1) and HTLV-2 Env (Env-2) show high sequence homology in their SU and TM subunits, with 65% and 79% residue conservation, respectively (Figure 1) [70]. Env-1 and Env-2 both require a combination of molecules on the cell surface as receptors for entry but utilize slightly different complexes. Env-1 utilizes heparan sulfate proteoglycans (HSPGs) and neuropilin-1 (NRP-1) for attachment and binding, and glucose transporter-1 (GLUT-1) for entry [69,71,72,73]. Env-2 uses GLUT-1 and NRP-1 for both binding and entry [71,73], but not HSPGs [74]. In addition, although they are highly similar in sequence and receptor usage, mutagenesis of equivalent functional regions in the subunits of Env-1 and Env-2 has revealed slight phenotypic differences in SU glycoproteins, as measured by quantitative assays for precursor cleavage, SU shedding into the supernatant, syncytium formation, and infectivity [70]. In contrast, the functional domains of TM were strictly conserved between HTLV-1 and HTLV-2 and were demonstrated to be critical for fusion and cell-to-cell transmission of both viruses. This emphasized the link between the structure and function of Env glycoproteins and suggested that SU subunits of HTLV-1 and HTLV-2 differ in the conformational information that they relay to TM to trigger fusion.

## 3. Distinct Transformation Preferences of HTLV-1 and HTLV-2

As its entry receptors are ubiquitously expressed, HTLV is capable of establishing infection in various cell types in vitro. Cells of the nervous system [75,76], endothelial cells [77,78], monocytes [76,79], and B-cells [79] are susceptible to infection; however, only T-cells undergo HTLV-mediated immortalization and transformation [80]. Despite their distinct in vivo pathogenesis, both HTLV-1 and HTLV-2 immortalize T-cells in culture and, intriguingly, exhibit preferences for specific subsets. HTLV-1 preferentially targets CD4^+^ T-cells, whereas HTLV-2 targets CD8^+^ T-cells [81]. These in vitro data are consistent with in vivo observations, as HTLV-1 is found preferentially in CD4^+^ T-cells—both in asymptomatic carriers as well as in patients with leukemia or neurological conditions [82,83,84]. It has also been demonstrated in vitro that Tax-mediated HTLV-1 transcription is significantly enhanced in purified CD4^+^ compared to CD8^+^ T-cells, suggesting that amplified viral transcription drives cell tropism and leukemogenesis mediated by HTLV-1 [85]. Nonetheless, studies in HAM/TSP patients have reported an increased HTLV-1 proviral load and preferential expansion of HTLV-1 Tax-specific CD8^+^ T-cells in the cerebrospinal fluid, demonstrating that an additional HTLV-1 reservoir can be established in CD8^+^ T-cells in vivo [86].

HTLV-2 has been found in both CD4^+^ and CD8^+^ T-cell subsets in vivo [87,88,89], although the proviral burden is higher in CD8^+^ T-cells [88]. To confirm the tropism of HTLV-2 in vitro, Wang et al. investigated the ability of the virus to infect and transform purified CD4^+^ or CD8^+^ T-cells in an established HTLV experimental system [90]. In the context of this experimental system, immortalization is defined as continuous cellular proliferation with the addition of exogenous interleukin-2 (IL-2) to the culture medium, while transformation is defined as IL-2-independent proliferation [80]. As HTLV replicates poorly in cell culture compared to other retroviruses and requires cell-to-cell contact for efficient infection [52,53,54,55], the in vitro immortalization assay is a widely accepted system used within the field to study early infection events [80]. To assess susceptibility to the virus and the replication pattern of HTLV-2 in each T-cell subset, virus producer cells were lethally irradiated and co-cultivated with CD4^+^ or CD8^+^ T-cells isolated from healthy donor peripheral blood mononuclear cells (PBMCs). HTLV-2 was shown to equally infect both purified CD4^+^ and CD8^+^ T-cells, and there were no observed differences between the cell types in terms of viral transcription or viral particle production, as measured by the detection of p19*^gag^* antigen in the culture supernatant. However, CD8^+^ T-cells were preferentially transformed when the HTLV-2 producer cells were co-cultured with a mixed population of PBMCs or purified T-cell subsets. These early experiments indicated that further study of the genetic differences between HTLV-1 and HTLV-2 could reveal the viral determinant of transformation tropism and suggested that these preferences could be driven by interactions between viral proteins and cellular proteins specific to each T-cell type.

**Figure 2:**
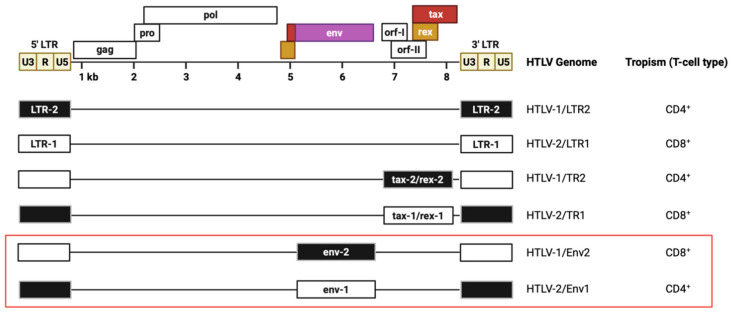
In vitro immortalization tropism of HTLV-1 and HTLV-2 recombinant proviral clones. Organization of the HTLV genome is depicted schematically, including the *gag*, *pro*, *pol*, *env*, *tax*, and *rex* genes in their respective reading frames, as well as *orf-I* and *orf-II*. For the recombinant proviral clones below, the altered region is indicated by white boxes (HTLV-1 origin) or black boxes (HTLV-2 origin), with the genetic background specified to the right. The immortalization tropism of CD4^+^ or CD8^+^ T-cells is shown for each virus. The red outline highlights recombinants which show a shift in T-cell tropism compared to the parental, wild-type viruses.

Follow-up studies sought to narrow down the genetic region of HTLV-1 and HTLV-2 that confers their distinct transformation preferences. An initial focus was placed on Tax, given its strong expression early during infection and its ability to activate both viral and cellular transcription [80]. The *tax* gene is located in a reading frame that is separate from, but overlaps with, the reading frame carrying the *rex* gene, whose product regulates the transport of unspliced and incompletely spliced viral mRNAs into the cytoplasm [91,92]. Ye et al. conducted the first study using recombinant molecular clones of HTLV-1 and HTLV-2 and exchanged the *tax* and *rex* genes between the viruses to determine the effect on transformation tropism (Figure 2) [93]. The recombinant viruses showed altered functional activity of Tax and Rex compared to wild-type clones. However, when producer cells carrying the recombinant proviruses were co-cultured with PBMCs in vitro, they retained the capacity to immortalize primary T-cells similar to the wild-type parental viruses. HTLV-1 proviruses encoding HTLV-2 *tax/rex* immortalized CD4^+^ T-cells in cell culture, while HTLV-2 proviruses encoding HTLV-1 *tax/rex* immortalized CD8^+^ T-cells. These findings suggested that *tax/rex* are not responsible for the diverging in vitro immortalization/transformation tropism of HTLV-1 and HTLV-2.

## 4. Role of Env in HTLV T-Cell Transformation Tropism

Experiments with HTLV-1 and HTLV-2 *tax/rex* recombinants have suggested that other viral sequences or elements dictate the transformation preferences of these viruses. Xie et al. generated additional mutants with the LTR or *env* gene exchanged between HTLV-1 and HTLV-2 to determine the contributions of these sequences [94]. The *env* gene recombinants, but not viruses with LTR sequences exchanged, showed a shift in transformation tropism compared to the parental viruses. Env-1 in an HTLV-2 genetic background (HTLV-2/Env-1) and wild-type (wt)HTLV-1 transformed CD4^+^ T-cells, while HTLV-1/Env-2 and wtHTLV-2 transformed CD8^+^ T-cells. This was the first study to highlight a potential role for Env in the pathogenic differences between HTLV-1 and HTLV-2. The observed preferences for T-cell type in vitro also have clinical relevance. HTLV-1-associated ATL is a CD4^+^ T-cell malignancy, while a higher proviral burden in CD8^+^ T-cells has been found in HAM/TSP patients and asymptomatic HTLV-1 carriers [95,96,97]. HTLV-2-infected individuals also show higher proviral burden in CD8^+^ T-cells [97]. In vitro data combined with observations from patients suggest that CD4^+^ and CD8^+^ T-cells are key players in the mechanisms underlying HTLV-mediated disease development.

To determine whether the distinct transformation tropism conferred by viral Env is at the level of entry or occurs later during infection, Kannian et al. investigated preference for T-cell type in an immune competent New Zealand white (NZW) rabbit model of HTLV infection and persistence [43,81,98,99,100,101,102,103]. Although they do not develop disease, rabbits inoculated with lethally irradiated HTLV-1 or HTLV-2 producer cells become persistently infected [43,99]. Given that HTLV establishes persistent, asymptomatic infection for variable lengths of time prior to detection in patients, the rabbit model allows for the analysis of T-cell tropism during initial infection. Peripheral blood was collected from HTLV-1 or HTLV-2-infected rabbits at weekly time points, and proviral load was determined by PCR over the 12-week study in CD4^+^ and CD8^+^ T-cells isolated from blood. The data showed that both cell types are infected as early as week 1 post-inoculation with HTLV-1 or HTLV-2, and there is no T-cell preference exhibited by either virus at the initial stages of infection in vivo [81]. This in vivo analysis demonstrated that there is no distinct entry tropism exhibited by HTLV-1 and HTLV-2, and that viral Env may drive transformation tropism at a later stage of infection [81]. To evaluate the CD4^+^ and CD8^+^ phenotype of HTLV-transformed T-cells longitudinally, the in vitro immortalization assay was conducted over a 9-week period. In the early weeks, CD4^+^ and CD8^+^ T-cells grew proportionally in HTLV-1 or HTLV-2 co-culture conditions; however, over time, the preferred subset emerged as a dominant population [81]. These data suggest that transformation tropism is driven by post-infection events during selective T-cell clonal expansion. They also reflect the long clinical latency period of HTLV-1 preceding the development of malignancy in patients and align with the current model of viral spread through cell division rather than de novo infection [34].

While NZW rabbits mimic asymptomatic infection in humans and enable the study of early viral replication patterns, they are not a suitable model for HTLV-mediated disease development. Huey et al. examined the contribution of Env to T-cell transformation in humanized mice used to model lymphoproliferative disease induced by HTLV infection [104]. Humanized mice were generated by injecting human umbilical cord stem cells into the livers of sub-lethally irradiated NSG mice, which reconstituted their immune systems with human lymphocytes that are phenotypically normal but unable to mount an adaptive immune response. Infection of the mice with wild-type HTLV-1/2 viruses resulted in preferential T-cell transformation (CD4^+^:HTLV-1 and CD8^+^:HTLV-2). Recombinant HTLV-2/Env-1 drove proliferation of CD4^+^ T-cells, while HTLV-1/Env-2 drove proliferation of both CD4^+^ and CD8^+^ T-cells. In contrast to their distinct pathogenic outcomes in humans, both HTLV-1 and HTLV-2 induced leukemia/lymphoma in mice, emphasizing the importance of immune function in limiting infection and disease progression [104]. Although transformation tropism could not be attributed directly to the *env* gene, this study demonstrated the utility of humanized mice as a model for HTLV T-cell preference and lymphoproliferative disease, and supports the investigation of how other viral proteins, such as Tax and Hbz, function together with Env to drive cellular transformation and oncogenesis.

It has been posited that differences in the in vitro and in vivo T-cell tropism of HTLV-1 and HTLV-2 can be ascribed to different receptor usage by these viruses. CD4^+^ T-cells, favored by HTLV-1, express higher levels of HSPGs (utilized by Env-1 for attachment to target cells) compared to CD8^+^ T-cells [69,72,74]. CD8^+^ T-cells, favored by HTLV-2, express more GLUT-1 compared to CD4^+^ T-cells [74]. HTLV-1 and HTLV-2 SU have been shown to exhibit higher levels of binding and entry on CD4^+^ and CD8^+^ T-cells, respectively. In transfection experiments to alter the levels of these receptors on the cell surface, GLUT-1 overexpression enhanced the entry of HTLV-2 virions into CD4^+^ T-cells, and HSPG expression on CD8^+^ T-cells increased HTLV-1 entry [74]. These results indicate that preferential tropism may be partially guided by diverging receptor requirements of these viruses. Nevertheless, these receptors are ubiquitously expressed, and although infection can be established in a wide variety of cell types [75,76,77,78,79], only T-cells are susceptible to HTLV transformation. Moreover, HTLV-1 and HTLV-2 can be detected equivalently in both T-cell subsets during initial infection in vivo, and during in vitro immortalization, CD4^+^ and CD8^+^ T-cells proliferate comparably, until one population is selected for predominant clonal expansion [81]. This evidence suggests that post-entry mechanisms drive the selection and outgrowth of the preferred cell type.

## 5. Role of Env in the Pathogenesis of Other Retroviruses

Studies of other oncogenic retroviruses have shown that Env can contribute to viral transforming activities. Env of Friend spleen focus-forming virus (SFFV) interacts with the erythropoietin receptor to induce the proliferation and differentiation of erythroid cells in the absence of erythropoietin [105]. This was found to occur through the interaction of the Env glycoprotein with a form of the Stk receptor tyrosine kinase unique to erythroid cells. It was also shown that co-expression of SFFV Env with this form of Stk could induce deregulated growth of non-erythroid cells [106]. For the simple retrovirus Jaagsiekte sheep retrovirus (JSRV), expression of Env alone is sufficient to transform a variety of cells in culture [107]. In vivo, an adenovirus-associated JSRV Env expression vector inoculated into immunodeficient mice has been shown to lead to the development of lung adenocarcinoma [108], and a replication-defective JSRV virus only expressing LTR-driven Env upon integration was capable of inducing ovine pulmonary adenocarcinoma lesions in experimentally infected lambs [109]. The cytoplasmic tail of the TM subunit contains an interaction site for phosphatidylinositol 3-kinase (PI3K), which may indirectly contribute to cell transformation mediated by JSRV Env [107]. In addition to both the PI3K-dependent and -independent Akt pathways, other major pathways have been implicated in this transformation process—including MAPK, as well as signaling involving the Hyal2 cell entry receptor-RON receptor tyrosine kinase [107]. Several studies have also investigated the role of the SU subunit of JSRV Env in transformation. While it has been shown that exchanges of the receptor-binding domain and proline-rich region between JSRV Env and Moloney murine leukemia virus has little effect on 208F cell transformation [110], SU mutants with sequence deletions or small insertions lose the capacity for transformation of this cell type and NIH 3T3 cells [111]. In the latter study, co-transfection of 208F cells with transformation-defective mutants of the SU and TM could rescue Env-mediated transformation, suggesting that both SU and TM domains may contribute to transformation [111]. For HTLV, single amino acid substitution in the surface domain of HTLV-1 Env at residue 195 shifts transformation tropism from CD4^+^ to CD8^+^ T-cells in culture [112]. Consistent with the data from recombinant HTLV-1 and HTLV-2 Env mutants, wtHTLV-1 and the N195D mutant virus functioned similarly with respect to the binding and entry of CD4^+^ T-cells. The predominance of CD8^+^ T-cells developed more slowly compared to wtHTLV-2, suggesting that the transformation tropism of HTLV results from the cooperation of other domains or residues [112].

## 6. Immune Response to Env

HTLV-1 infection elicits both humoral and cell-mediated immune responses. Infants born to HTLV-1-positive mothers are protected from infection in their first months of life through transplacental acquisition of anti-HTLV-1 maternal antibodies [113]. Anti-HTLV-1 antibody titer in an individual correlates with their proviral load, a measure of viral burden defined as the number of HTLV-1 proviral copies per 100 PBMCs [114]. Other major contributors to the immune response against HTLV-1 are cytotoxic T-cells (CTLs), most of which are specific to the Tax protein [115,116,117,118]. Since high proviral load is a risk factor for both ATL and HAM/TSP [32,119], CTL deficiency may serve as an additional distinctive marker for disease development [120].

Alongside Gag and Tax, Env is one of the immunodominant viral proteins [114], as it is required for the entry of target cells and establishment of initial infection. Using sera from HTLV-1-infected patients, neutralizing antibody responses have been mapped to epitopes within the SU [121,122]. Mice immunized with a soluble, recombinant SU fused to the human IgG Fc region have exhibited high antibody titers, and monoclonal antibodies to the SU from the mice were shown to block Env-mediated receptor binding and viral entry into cells [123]. In addition, experimental vaccines utilizing Env as an immunogen have exhibited partial protection against HTLV-1 challenge in animal models [124,125]. Vaccinia virus-derived vectors encoding the *env* gene and peptide constructs containing a B-cell epitope from the Env region conferred protection to immunized non-human primates challenged with HTLV-1 [124,125].

## 7. Contributions of Other HTLV Genes to Transformation

### 7.1. Tax 

Tax is the key regulator of HTLV gene expression and the primary driver of the cellular transformation process. Tax functions in transcriptional activation involving major players such as NF-κB, SRF, and CREB, and influences signal transduction, apoptosis inhibition, dysregulation of the cell cycle, and disruption of tumor suppressors [34,126]. The presence of functional Tax is essential for transformation of primary human T-cells mediated by HTLV-1 and HTLV-2 [127,128]. However, numerous differences have been characterized in the interactomes and activities of HTLV-1 Tax (Tax-1) versus HTLV-2 Tax (Tax-2), such as differential activation of NF-κB, effects of post-translational modifications, and cellular localization [60,129,130]. These factors are probable contributors to the divergent pathogenic outcomes of HTLV-1 and HTLV-2 infection. Moreover, enhanced viral transcription is mediated by Tax-1 in purified CD4^+^ compared to CD8^+^ T-cells, which likely potentiates leukemogenesis by HTLV-1 [85]. Due to its high immunogenicity [29,45], however, Tax cannot be expressed continuously. Selection is directed toward cells with minimal Tax expression due to the potent CTL response against the protein [34]. However, the CTL response in patients is chronically activated by the recognition of sense-strand transcripts—particularly Tax [115]. This is suggestive of a transient activation of viral transcription and Tax expression in vivo. Recent studies have demonstrated HTLV-1 transcriptional bursts in PBMCs from infected patients cultured ex vivo [131]. Additional studies have demonstrated that spontaneous on/off switching of Tax in subpopulations of HTLV-1-infected leukemic cells maintains the survival of the population as a whole through the induction of anti-apoptotic signals [132]. ATL cases are heterogenous and can be categorized into subtypes where Tax expression is high, intermittent, or lost entirely [133]. This intricate biological system of inducible Tax has emerged as a critical immune evasion strategy to promote the persistence and leukemogenic potential of HTLV-1 infection. Although Env is one of the immunodominant proteins of the virus, and Env-specific antibody responses can be detected in ATL, HAM/TSP, and asymptomatic cases [134], it is continuously expressed during in vitro immortalization of T-cells [100]. Moreover, it is genetically stable and highly conserved among viral isolates both in its nucleotide and amino acid sequences [135]. These aspects may favor Env as a therapeutic target or a vaccine candidate in the prevention of HTLV-1-associated disease, but further studies to dissect the interactions of Env in CD4^+^ and CD8^+^ T-cells are required. While Env is detectable during early immortalization in vitro, viral sense transcription is silenced in vitro and in vivo through mechanisms such as 5′ LTR hypermethylation or deletion [136,137,138,139]; therefore, the contributions of Env to T-cell immortalization may occur during the early stages following initial infection, as it is likely remains inactive during HTLV-1 latency.

### 7.2. Hbz

In contrast to Tax, Hbz expression is continuous in HTLV-1-infected individuals [34]. It has been shown that *hbz* mRNA promotes T-cell proliferation and Hbz protein inhibits viral transcription by Tax [41]. Although dispensable for viral replication and cellular immortalization mediated by HTLV-1 in vitro, Hbz contributes to viral persistence in the in vivo NZW rabbit model [43]. Rabbits infected with HTLV-1 proviruses deficient for Hbz protein, but not RNA, were able to establish infection, but had decreased proviral load and HTLV-1-specific antibody responses. Short hairpin (sh)RNAs effective in knocking down *hbz* mRNA and Hbz protein expression in established HTLV-1 T-cell lines and newly immortalized HTLV-1 T-cells significantly reduced their proliferation in culture [42]. Engraftment of shRNA-mediated Hbz knockdown SLB-1 cells (HTLV-1 transformed cells) into NOD/SCID^γc−/−^ (NOG) transplant mice resulted in significant decreases in tumor formation and infiltration into organs compared to that observed with scramble SLB-1 cells [42]. These experiments confirm the role of Hbz in maintaining the proliferative and tumorigenic capacities of HTLV-1-infected cells. In addition, Hbz counteracts many of the functions of Tax through suppression of NF-κB, AP-1, and viral gene expression by sequestering CREB and CBP/p300 from binding to the viral cyclic AMP response elements within the 5′ LTR [40]. Notably, *hbz* mRNA does not trigger a CTL response, revealing another key HTLV immune evasion mechanism [40]. The HTLV-2 counterpart of Hbz, Aph-2, is dispensable for in vitro immortalization and persistent infection in NZW rabbits [99]. Inoculation with an Aph-2 knockout virus, however, resulted in a corresponding increase in proviral load and antibody response in the rabbits during early infection. Additional comparative studies of Hbz and Aph-2 have shown that they have divergent functional roles in modulating cellular pathways [47]. Differences in several viral genes between HTLV-1 and HTLV-2 are likely to account for the distinct in vivo pathogenesis of these viruses. Characterizing the divergent functions of HTLV-2 proteins will highlight features that suppress disease development during HTLV-2 infection. Importantly, since ATL onset and progression is a multi-step process, no viral gene is sufficient to cause disease; rather, it is the accumulation of genetic and epigenetic changes that drives leukemogenesis [38].

## 8. Conclusions and Future Directions

Findings from HTLV transformation tropism studies hold clinical relevance, as the in vitro immortalization assay mimics natural cell-to-cell transmission and recapitulates in vivo tropism observed in patients. This experimental system continues to be a useful tool to examine nuances in the molecular biology of HTLV-1 and HTLV-2—two closely related, yet pathologically distinct viruses. Future studies are needed to address the functional differences in Env-1 and Env-2 beyond viral entry, in the context of CD4^+^ and the CD8^+^ T-cell environments, which may contribute to the distinct transformation preferences of HTLV-1 and HTLV-2. These studies will uncover Env-driven post-entry events which mediate the selection of a particular T-cell type for predominant clonal expansion or define specific regions or residues of Env protein that confer these activities. They may also identify synergistic effects with other viral proteins. Does Env set the stage for Tax and Hbz in the immortalization of HTLV-1-infected cells? Does Env, in concert with Hbz, help drive cellular proliferation and mitotic viral replication in the absence of Tax expression? The phase of the immortalization/transformation process in which Env takes part is yet to be elucidated. Additionally, it is possible that further study of Env-2 or other HTLV-2 proteins will provide clues to the long clinical latency period of HTLV-1 infection. Dissecting similarities and differences in the timing and level of gene expression or protein function and interactions with the cellular environment that allow HTLV-2 to persist, but not progress to disease, may illuminate parallels to HTLV-1 latency.

## Data Availability

Not applicable.
